# A systematic review exploring the role of tuberculosis stigma on test and treatment uptake for tuberculosis infection

**DOI:** 10.1186/s12889-024-20868-0

**Published:** 2025-02-14

**Authors:** Ayşenur Kılıç, Xuanyu Zhou, Zoe Moon, Yohhei Hamada, Trinh Duong, Charlotte Layton, Sobhash Jhuree, Ibrahim Abubakar, Molebogeng X. Rangaka, Robert Horne

**Affiliations:** 1 School of Pharmacy, University College London, London, WC1H 9JP UK; 2 Institute for Global Health, University College London, London, UK; 3 MRC Clinical Trials Unit, University College London, London, UK; 4 NOCLOR NHS Research Office, London, UK; 5 Division of Epidemiology and Biostatistics & CIDRI-AFRICA, University of Cape Town, Cape Town, South Africa

**Keywords:** Tuberculosis, TB, Infection, Adherence, Test and treat, Prevention

## Abstract

**Background:**

Tuberculosis (TB) stigma may be a barrier to engagement in testing and treatment for TB infection (TBI). We systematically reviewed the available evidence on how TB stigma influences engagement with TBI testing and treatment.

**Methods:**

Electronic databases (e.g., CINAHL, Central, OVID) were searched from 1963 to 1st August 2024. Quantitative, qualitative, and mixed-method studies reporting the effects of TB stigma on engagement with TBI testing and treatment were included in the review. Descriptive synthesis was applied to the quantitative studies, and thematic analysis was applied to qualitative studies. The risk of bias was assessed by using the mixed methods appraisal tool.

**Results:**

Seventeen studies were included in the review (12 qualitative, four quantitative and one mixed methods). TB stigma was complex and multifactorial with six overlapping domains: public, anticipated, self, experienced, secondary, and structural. Perceptions or experiences of stigma were associated with lower rates of engagement in testing and adherence to treatment in TBI.

**Conclusions:**

Perceptions of TB stigma among people with TBI were related to the common social representation of TB disease such as its being contagious or disease of the poor. Negative perceptions of active TB appear to carry over to its infection, despite people being informed about the nature of TBI. Our findings could inform more effective communication to support TBI testing and treatment engagement.

**Supplementary Information:**

The online version contains supplementary material available at 10.1186/s12889-024-20868-0.

One-quarter of the world’s population carries Mycobacterium tuberculosis antigens without clinical manifestations of tuberculosis (TB) disease, a condition known as tuberculosis infection (TBI), or ‘latent TB’ [1, 2]. It is estimated that without treatment, approximately five per cent of people with TBI will develop active TB, with the risk being even higher in some groups or settings (e.g., people with human immunodeficiency virus [HIV] co-infection, suppressed immunity, or with limited access to health care) [2, 3]. Testing and treating TBI is therefore a crucial epidemic control strategy, to suppress the spread of TB within communities and decrease the global burden of TB disease [3]. However, TBI testing and treatment rates are suboptimal, with rates falling below 90% for both, as recommended by the World Health Organisation (WHO) [4, 5]. For instance, a systematic review and meta-analysis (*k* = 58) focusing on the attrition of people throughout the testing and treatment stages of TBI reported significant losses occurring during the initial testing (71,9%, *95% Confidence Internal [CI]* 71.8–72.0), completion of the medical evaluation (47.3%, *95% CI* 42.5–44.9), and the initiation (30.7%, *95% CI* 26.8–32.1) and completion (18.8%, *95% CI* 16.3–19.7) of TBI treatment [6]. Thus, understanding the barriers to engagement with testing or treatment procedures for TBI control is an essential target to prevent active TB cases and their spread within communities [3]. 

In 2022, the Global Fund Strategy identified TB-related stigma as a potential barrier to achieving the WHO END-TB strategy and identified reducing TB-related stigma as one of its goals for the 2023 to 2028 period [7]. Stigma is a complex and multidimensional psychosocial process where a person’s differing characteristics (e.g., gender) or experiences (e.g., medical condition) diverge from social norms, leading others to devalue, label, discriminate, isolate, or exclude the person from the group [8]. Certain health conditions and illnesses may be perceived negatively by the public, and this can result in individuals who are diagnosed with the condition perceiving that they are devalued by others as a result of this [8, 9]. This may be reinforced if people or institutions are seen to behave in a different way that is perceived by the individual to be discriminatory; known as health-related stigma [9]. 

TB is associated with health-related stigma, often linked to perceptions of TB as a disease (e.g., infectiousness and incurability); [10, 11] but, also related to negative perceptions about the type of person that gets TB (e.g., low social class) [11]. The TB stigma is conceptualised with its various subtypes, which may either interact with each other or co-occur [12]. Initially, stigma could be driven by the negative perceptions of the public towards TB, which is also known as public stigma [12] (e.g., shared belief to avoid or exclude people with TB). Anticipated stigma occurs when individuals’ awareness of negative perceptions towards TB results in fear of being labelled or devalued due to their association with TB (e.g., being afraid of being associated with TB due to fear of exclusion) [12]. Further, individuals may internalise these negative perceptions as a result of their association with TB, viewing themselves through stigmatising labels, known as self-stigma (e.g., perceiving one’s TB status as a result of their bad deeds) [12]. Moreover, when these negative perceptions around TB lead to actions such as isolation, it becomes enacted stigma (from the perspective of the actor) or experienced stigma (from the perspective of the person affected, e.g., avoiding someone with TB) [12]. TB stigma can also be extended to individuals who may not have TB but may be associated with someone with TB and they may experience negative perceptions or behaviours from others, which is known as secondary stigma (e.g., marginalising health care practitioners [HCPs] who work with TB patients) [12]. Lastly, where laws or policies disadvantage individuals who are associated with TB, it becomes a structural stigma (e.g., heavily testing one ethnic group while other communities may have a similar chance of being affected by TB) [12]. 

Evidence suggests that people experiencing active TB symptoms often refrain from seeking healthcare due to the stigma associated with TB [13]. People with TB also report being avoided or excluded from socioeconomic opportunities (e.g., loss of employment) [11] due to fear of being exposed to TB. It is anticipated that similar challenges will arise when attempting to engage individuals with TBI testing or treatment, as people may not differentiate between active TB and TB infection, with perceptions like TB being infectious and symptomatic also being associated with TBI [14]. Therefore, it is important to understand the perspectives of the person who is experiencing TBI and others around them (e.g., family members) to provide the full picture of TB stigma and its impact on testing and treatment engagement. The chance of experiencing TB stigma may negatively influence someone’s perceptions to attribute less importance towards TBI care due to potential implications of TB stigma (e.g., social exclusion) resulting in not taking part in TBI testing and treatment [15]. Further, the need to take regular medication, possible side effects or dietary restrictions (e.g., not drinking alcohol) linked to the treatment make it harder for people to conceal their diagnosis from others [14]. Thus, increasing concerns towards the TBI testing or treatment.

Whilst previous reviews have explored the effect of TB stigma in active TB disease or mixed TB populations (active TB and TBI) [11, 16, 17], no previous systematic review has explored the effect of TB stigma on TBI testing and treatment engagement. This systematic review explores the role/effect of stigma in the uptake and engagement with TBI diagnostic testing and treatment among at-risk communities. The research questions are defined as follows:

1. What is the nature of stigma around TBI within TBI testing and treatment?

2. What is the role/effect of TB stigma on taking part in each step of TBI testing activities (e.g., taking the TBI test or attending results reading) among TB-risk communities, who are invited to take the TBI test due to their higher risk of TB infection?

3. What is the role/effect of TB stigma on treatment adherence (initiation, implementation, and discontinuation) among people diagnosed with TBI?

## Methods

### Protocol and registration

This review protocol is registered on PROSPERO: CRD42023396674 (Version 1, 21.04.2023) and can be found online. This systematic review was conducted and reported based on the guidelines set by the Cochrane Handbook for Systematic Reviews of Interventions [18], and the Preferred Reporting Items for Systematic Reviews and Meta-Analyses (PRISMA) statement [19]. Evidence synthesis was enhanced by following the guidance developed by the JBI Mixed Methods Review Methodology Group for combining the quantitative and qualitative data [20]. 

### Eligibility criteria

Studies were included if people were at risk of TBI and progression to disease who are eligible for or offered TBI testing (e.g., people with social and clinical risk factors of homelessness, drug and/or alcohol problems, HIV status) or TBI treatment from any gender and age, with no geographic restrictions. Caregivers or HCPs reporting the impact of TB stigma on the care recipient’s testing and treatment of TBI were also included in the review. Quantitative, qualitative and mixed methods studies reporting TB stigma are included in this review (Table 1 for the structure of reporting stigma for included studies in this review).

Studies were excluded if participants were diagnosed with active TB or not eligible for TBI testing or treatment, such as low-risk communities. Further, participants who experienced any other type of health condition-related stigma, such as HIV only without linking to TBI testing and treatment, and TB stigma itself were excluded from the study. Studies without full reports (e.g., conference abstracts), editorial letters, and systematic reviews and meta-analyses were excluded from the review.

**Table 1:** Structure of reporting stigma for the studies included in this review

Qualitative studies	Quantitative studies
i) Any TBI-related stigma (e.g., anticipated, experienced or internalised[self]), excluding secondary stigma (stigma experienced due to being associated with someone who experiences TB stigma [12] linked to research questions of this systematic review and explored as a theme or discussed in the research findings. Studies with secondary stigma on TBI will only be included if its effects are linked to the experiences of people with TBI and study outcomes	i) Any standardised measure of stigma or perceived/internalised stigma if linked to TBI
ii) Discrimination or perceived poor treatment due to being linked to TBI (e.g., suitable people for TBI testing) or one’s TBI status	ii) Any measure of attitudes to people with TBI reporting the outcomes related to this systematic review questions
iii) Intersectional stigma (e.g., gender, race) or any other health-condition-related stigma (e.g., HIV-related stigma) will be included in the review if this is linked to TBI	iii) Any measure of intersectional stigma or other health condition-related stigmas which are linked to TBI

### Search methods for identification of studies

***Electronic searches*** Seven electronic databases were searched: the Cochrane Central Register of Controlled Trials (Central), the OVID databases (Medline, Embase, PsychINFO, Global Health, and Web of Science), and the Cumulative Index to Nursing and Allied Health Literature (CINAHL) Plus. “Tuberculosis” and “stigma” terms with equivalent MeSH terms (medical subheadings) and their synonyms were used in the search strategy (Appendix A for detailed search strategy). Searches were carried out from 1963, when Erving Goffman’s seminal work viewed as the start of stigma research [8] to 1st August 2024.

***Searching the grey literature*** Grey literature was identified by searching PhD theses and dissertations via EThoS, and hand-searching the literature of the included papers to identify suitable papers.

### Data collection and analysis

***Selection of studies*** Two independent researchers (AK & ZZ) conducted the literature searches following the pre-determined search strategy in the selected databases from 1963 to 1st August 2024. Further, grey literature was searched independently by both reviewers. Duplicate papers were removed in three steps: (i) identified papers were exported to EndNote Reference Management Software [21], and removed by its ‘find duplicates’ function; (ii) then the rest of the papers exported to the Rayyan web app [22] for carrying out systematic review data selection and its artificial intelligence (AI) supported duplicate removal system were used, (iii) lastly the hand search was used for removing the duplicates. Once all duplicates were removed by using the Rayyan platform, the papers’ titles and abstracts were screened against the pre-defined inclusion/exclusion criteria by two independent researchers (AK & ZZ). Then, papers passing the initial title and abstract screening were included for full-text review by using a prior screening table. The number of in/eligible papers was recorded by using a PRISMA flow diagram. The findings of both reviewers were compared, and discrepancies were resolved by discussion. Reviewers’ agreement assessed by Cohen’s Kappa agreement [23] for 10% of randomly selected papers included in the review.

***Data extraction and management*** The findings of the included papers were summarised by using a predetermined data extraction table by two independent reviewers. The data extraction table included information on the study, sample, the type of stigma, study findings and limitations. Further, the results of the qualitative studies were separately exported for each study and imported to NVivo Software [24] to summarise the recurring themes or patterns present in qualitative data by applying thematic analysis [25]. 

### Assessment of risk of bias in included studies

The quality of the included studies was assessed by using the mixed methods appraisal tool (Version 2018) for qualitative, quantitative, and mixed methods studies [26]. Further, the quality of the included papers was assessed by two independent reviewers for approximately half of the randomly selected papers to confirm their consistency.

### Data synthesis

The effect of TB stigma on: (i) diagnostic and (ii) treatment uptake or adherence for people affected by TBI is demonstrated with a descriptive synthesis. TB stigma and its subtypes, as explained earlier in the paper, were further explored in the included studies, where the author (AK) identified different types of TB stigma, unless the studies directly reported its subtypes, and explored their role/impact on the testing and treatment of TBI. Qualitative and quantitative results were separately summarised while also bringing out the overlaps and discrepancies among their findings. For mixed-methods studies, only findings relevant to our review objectives were considered. Lastly, the income levels of the included studies, as classified by the World Bank for the 2025 fiscal year [27], and the incidence rates of TB (e.g., low, medium, or high risk), as defined by the WHO in 2019 [28], were reported. The study results were not grouped by the level of TB incidence rates in the countries where the studies were conducted, due to an insufficient number of papers to perform such analyses.

## Results

### Description of studies

**Fig. 1:**
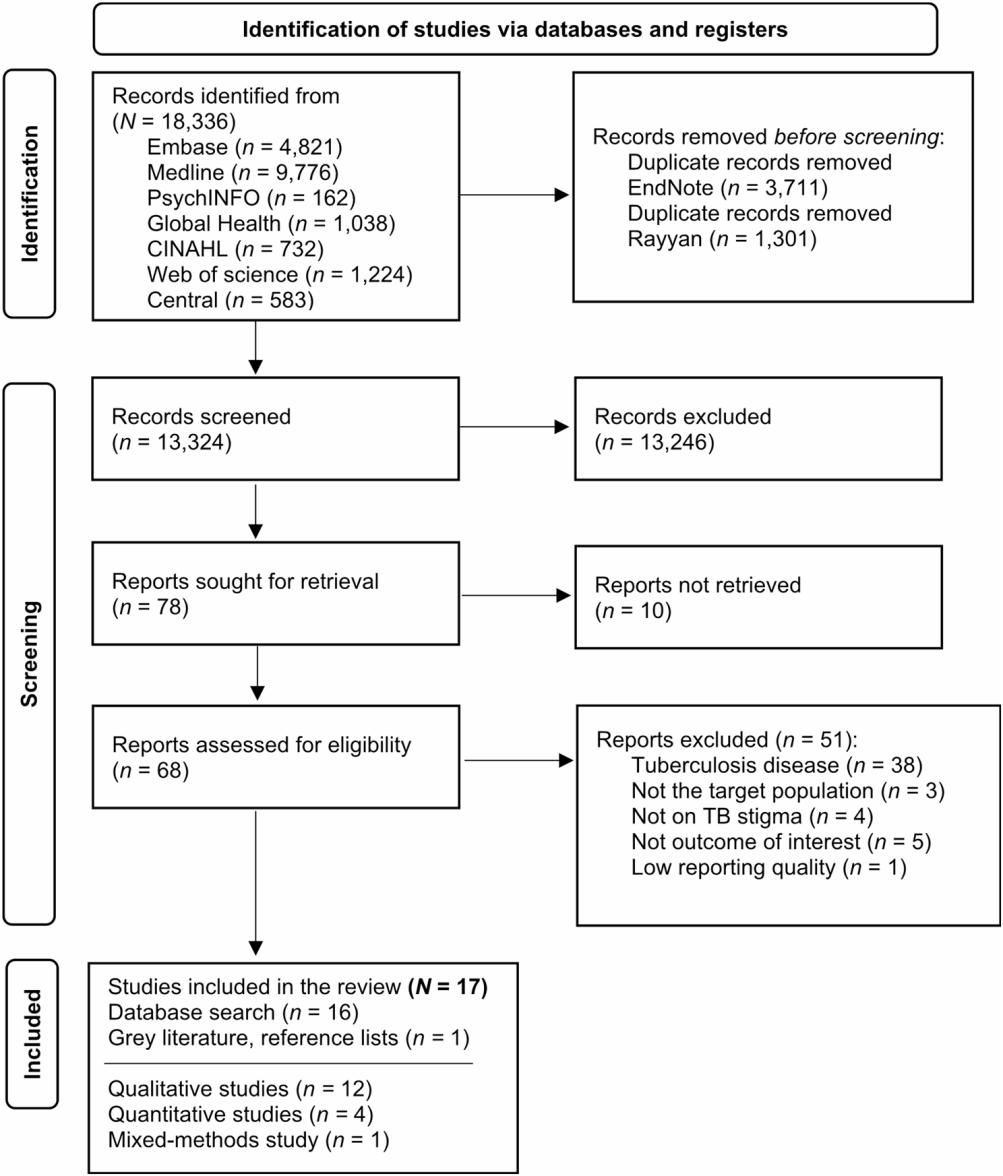
PRISMA flow diagram

***Results of the search*** Figure 1 PRISMA flow diagram summarises each step of the screening process. Reviewers had a substantial agreement (0.70) on the Kappa agreement [23] for 10% of randomly selected papers included in the review. In total, 18,336 records were screened, and 17 papers were found to be suitable for the systematic review.

***Included studies*** The overall findings of the included studies are summarised in Table 2. In total, 4,480 people were included in this review. The studies included people from the following countries: South Africa (*n* = 5), USA (*n* = 3), Canada (*n* = 2), Australia (*n* = 1), UK (*n* = 1), Botswana (*n* = 1), Dominic Republic (*n* = 1), Netherlands (*n* = 1), China (*n* = 1) and Uganda (*n* = 1). Age ranged from less than five years, where their carers were involved with the research, to 64 years, with nearly all of the reporting coming from adult (18 years old and older) participants. Most studies utilised the qualitative design (*n* = 12) followed by quantitative (*n* = 4), and mixed method (*n* = 1) designs to explore TB stigma. Overall, TB stigma was reported to be a barrier to engagement with TBI testing (*n* = 5) and treatment (*n* = 15). The following TBI high-risk groups (*n* = 16) were represented: new entrants from TB high-burden countries (*n* = 6, 37.5%), people living with HIV (*n* = 5, 31.25%), close contacts of people diagnosed with active TB disease (*n* = 2, 12.5%), people at occupational risk for TBI (*n* = 2, 12.5%), and people experiencing homelessness (*n* = 1, 6.25%). Outside of TBI risk groups, one study was conducted with college students.

**Table 2:** Description of included studies

Study, Year (Reference)	Country		Sample Characteristics	Study Design	Study aims and measures	Data analysis	Stigma subtypes	Results of included studies	Limitations
		TBI Testing Activities							
Hall, Kabir, Shih, & Degeling (2020)	Australia High income and low TB incidence		15 Indian and 13 Pakistani immigrants in Australia 12 (43%) males	Qualitative study: Semi-structured interviews	In-depth interviews exploring attitudes to TB of Indian and Pakistani immigrants living in the Illawarra-Shoalhaven area, and identify potential sociocultural, ethical and practical barriers to the uptake of targeted TBI testing by members of these communities	Thematic analysis	*Indirectly reported*: Public stigma, structural stigma	Participants reported TB/TBI diagnosis may bring in stigma and found targeting specific communities as an unfair burden imposed on those groups.	- Participants may not be fluent in English, despite the study material provided in English. - The findings of the study may not be applicable to populations beyond the scope of this specific group of participants.
Seedat, Hargreaves, & Friedland (2014)	UK High income and low TB incidence		20 participants from immigrant communities 10 (50%) males Mean age: 42.77 years Range: 25–64 years	Qualitative study: Semi-structured interviews	Semi-structured interviews exploring views around barriers, and accessibility of testing for TB and other conditions (e.g., HIV)	Constant comparative approach	*Indirectly reported*: Public stigma	Participants perceived that testing was not widely acceptable due to the stigma associated with the disease in their communities, as well as health services not being immigrant-friendly.	- Limited generalisability of the study findings.
		***Treatment Adherence***							
Gao et al. (2015)	Canada High income and low TB incidence		912 survey respondents 32.9% males The largest age range: 24–44 Focus groups: 1st: Two female and 4 male Chinese migrants with a mean age of 40.3 2nd: 6 female Chinese immigrants with a mean age of 34.5	Qualitative study: Two focus groups	Surveys and focus groups exploring the Chinese immigrants’ knowledge and perceptions of TBI	Descriptive summaries & conceptual mapping	*Indirectly reported*: Anticipated stigma, self-stigma	Participants reported fear of stigma and not wanting others to see them taking the medication every day due to associated stigma.	- The survey language was limited to English, despite recruiting from Chinese communities. - Focus groups may not have achieved saturation, especially for people living in rural areas of Canada. - Unable to collect views from non-participants.
Gust et al. (2011)	Botswana Upper middle income and endemic		Interviews: 18 participants took part in group discussions and 25 participants had individual discussions. Survey: 462 participants completed the surveys.	Mixed methods: A three-year clinical trial	Group and individual interviews, as well as a survey, were conducted to explore the factors influencing non-adherence among individuals who were offered IPT but rejected it or were lost to follow-up.	A grounded theory method analysis & descriptive summary	*Indirectly reported*: Experienced stigma	Stigma was identified as one of the barriers to trial participation during the interviews. Additionally, 2.4% of individuals in the non-adherent and lost to follow-up groups reported stigma associated with being in the trial as a self-reported reason for non-adherence.	- The possibility of bias exists due to self-reported measurements of non-adherence. - Some of the relevant variables regarding non-adherence may not have been included in the data collection.
Heyd et al. (2021)	Canada High income and low TB incidence		10 participants 6 (60%) males Mean age: 47 years Range: 17–64 years	A qualitative descriptive study: semi-structured interviews	Interviews to explore perceptions of TBI and its treatment among people offered isoniazid and rifapentine (3HP) while unstably housed or homeless.	Latent content analysis	*Indirectly reported*: Self-stigma	Participants reported experiencing stigma and disease-related shame as a barrier to TBI treatment access, uptake, and adherence. They hid the diagnosis from others.	- Small sample size - Recruitment challenges due to the transient nature of the study population.
Jacobson et al. (2017)	South Africa Upper middle income and severely endemic		17 completed IPT and 13 defaulters Completers’ median age: 39 years Defaulters’ median age: 37 years	A qualitative study: Semi-structured interviews	Interviews for exploring factors influencing IPT completion or default during HIV care.	Framework analysis	*Indirectly reported*: Anticipated stigma, HIV-related stigma	TB stigma compared to HIV stigma was not seen as a problem for treatment completion.	- Limited generalisability to other settings or HIV-positive patients who are not in the care yet
Kay et al. (2020)	USA High income and low TB incidence		40 adolescents living with HIV 21 (52.5%) males Mean age: 15.5	A cross-sectional survey study	A survey exploring the barriers to IPT uptake and completion in children and adolescents during HIV care.	Univariate logistic regression	*Indirectly reported*: Public stigma, anticipated stigma, HIV-related stigma	Primary caregivers who believed that taking a pill every day would lead to stigma from neighbours, thinking the child was sick or had TB, had increased odds of suboptimal adherence for the child (Odds Ratio [*OR]*: 2.46, *95% Confidence Interval [CI]*: 1.10, 5.51 and *OR*: 1.75, *95% CI*: 0.91, 3.37, respectively).	- Small cohort size likely to have low power. - The study sample had high rates of health-seeking behaviour limiting the findings’ generalisability.
Nathavitharana et al. (2021)	South Africa Upper middle income and severely endemic		22 participants 32% males	Qualitative study	Interviews exploring how healthcare workers perceived the occupational TB risk for testing and treatment.	Thematic analysis	*Indirectly reported*: Anticipated stigma	TB stigma is reported to impact one’s willingness to engage in care.	- The sample is limited to healthcare workers enrolled on the parent study.
Rowe et al. (2005)	South Africa Upper middle income and severely endemic		18 people with TBI and HIV 5 (27.78%) males Mean age: 35 Range: 20–60	Qualitative study	Interviews exploring barriers to TBI treatment among people with HIV.	Thematic analysis	*Indirectly reported*: Anticipated stigma, public stigma	A confidential clinic environment reduces patients’ fear of stigma and enhances one’s adherence.	- Over-representation of people who are users of the health services. - Adherence rates may be overestimated due to the use of pill counts or survey data.
Skinner, Hesseling, Francis, & Mandalakas (2013)	South Africa Upper middle income and severely endemic		2 caregivers providing IPT to their children and two TB clinic staff from each community	A qualitative study: In-depth interviews	Interviews discussing the knowledge and attitudes of parents to IPT administration to their children (< 5 years of age).	Interpretative content analysis	*Indirectly reported*: Anticipated stigma, HIV-related stigma	Caregivers reported needing to overcome stigma and threats of isolation from their neighbours to keep their children on IPT.	- Limited generalisability due to small sample size and inherence bias in sample selection.
Vásquez et al. (2022)	Dominic Republic Upper-middle income and lower moderate TB incidence		212 children (< 5 years of age) 104 (49.06%) males	Qualitative study	Semi-structured interviews to identify barriers and facilitators for IPT in children.	Content analysis	*Indirectly reported*: Self-stigma, structural stigma	People reporting being treated as equals, and not experiencing stigma, became more receptive to initiating treatment. Further, stigmatisation is also linked to one partner leaving the home and taking the children with them resulting in they cannot continue to the therapy.	- Study results need to be interpreted with caution due to the use of secondary sources of data collection.
Pekovic (1996)	Uganda Low income and endemic		Adults who were infected with HIV Cross-sectional survey (*n* = 251)	A randomised clinical trial of preventive TB therapy: cross-sectional survey	Establishing factors associated with compliance with preventive TB therapy	Multiple regression analysis	N/A	Stigma was not found to explain variance for compliance (*B*: 0.23, *p* = 0.30), even after controlling for socio-demographic factors and regimen-related characteristics.	- It covers various forms of stigma related to participation in a research study, HIV, and TB, without a specific focus on one type of stigma. - Possibility of survivor bias.
Williams (2015)	USA High income and low TB incidence		8 Burmese Chin refugees diagnosed with TBI 4 (50%) males Mean age: 33 years Range: 19–48 years	Qualitative study: Ethnography	An ethnographic exploration of Burmese Chin refugees’ experience of barriers to receiving TBI treatment.	Ethnographic analysis	*Indirectly reported*: Anticipated stigma, public stigma	Participants reported not wanting to disclose their TBI status to others and hid the medication-taking behaviour from others.	- Limited generalisability of the study findings. - The study was limited to a specific Chin sub-group.
Yuan et al. (2023)	China Upper-middle income and upper-moderate TB incidence		1547 college students diagnosed with TBI 846 (54.7%) males Mean age: 18.5 years	Cross-sectional analysis	A survey to explore the gender-specific association between perceived TB stigma and acceptance of TBI treatment among college students.	Multilevel mixed-effects logistic regression	*Directly reported*: Perceived stigma	Perceived TB stigma positively linked with acceptance of preventive treatment (*OR*: 1.03, 95% *CI* 1.00, 1.02; *p* = 0.005).	- Limited generalisability of the study findings. The study cannot demonstrate causality and only indicates correlation.
		***Joint Exploration of TBI Testing Activities & Treatment Adherence***							
Coreil, Lauzardo, & Heurtelou (2004)	USA High income and low TB incidence		Five focus groups, which included Haitian participants, with groups stratified by age and sex	Qualitative study: Focus groups	Group discussion on beliefs and practices related to TB/HIV testing	Content analysis	*Indirectly reported*: Anticipated stigma, public stigma, experienced stigma, structural stigma, HIV-related stigma.	Participants reported avoiding HIV/TB clinics due to the associated stigma. They felt anger when offered TB tests because they believed it singled them out based on their Haitian identity. Participants mentioned that the need to name contacts was seen as a potential barrier to seeking treatment.	- Participant characteristics were not reported in detail.
Sommerland et al. (2017)	South Africa Upper middle income and severely endemic		804 health care workers 221 (27.5%) males Mean age: 43.68	Cross-sectional survey that is part of a randomised controlled trial	A survey to explore whether the perception of stigma among healthcare workers influences their willingness to engage in TB services at the occupational health units.	Structural equation model	*Directly reported*: Other’s external stigma (secondary stigma)	Increased perceptions of other healthcare workers’ stigma in the workplace towards the use of occupational health units for TB decreased the probability of involving in testing (*B*: − 0.214, *p* = 0.000), treatment (*B*: -0.161, *p* = 0.001), and IPT (*B*: -0.170, *p* = 0.000).	- The analysis could not control for within- and between-variance effects between different hospitals due to the limited number of groups. - Alternative models, such as accessing occupational health care units outside of the hospital, were not explored.
Spruijt et al. (2020)	Netherlands High income and low TB incidence		26 Eritrean asylum seekers attended the group interviews regarding testing, while 31 Eritrean asylum seekers participated in individual interviews on TBI treatment.	Qualitative study: Semi-structured individual and group interviews	Semi-structured interviews exploring TBI-related knowledge, attitudes, beliefs and stigma among Eritrean asylum seekers and refugees.	Thematic analysis	*Directly reported*: Anticipated stigma, Experienced (enacted) stigma *Indirectly reported*: public stigma, HIV-related stigma.	Participants mentioned that experience of stigma was a barrier to their involvement in TBI testing and treatment.	- Unable to collect views from non-participants - The age and gender of study participants were not collected.

*Notes.* IPT: Isoniazid Preventive Therapy

Papers directly mentioning stigma subtypes are noted as **directly reported**; otherwise, they are noted as **indirectly reported**

High-income: $14,006 or more gross national income (GNI) per capita. Upper-middle income: $4,516 to $14,005 GNI per capita. Lower-middle income: $1,146 to $4,515 GNI per capita. Low-income: $1,145 or less GNI per capita [27]

Low TB incidence: less than 10 new and relapse cases per 100.000 population per year. Lower-moderate TB incidence: 10 to 49 new and relapse cases per 100.000 population per year. Upper-moderate TB incidence: 50 to 99 new and relapse cases per 100.000 population per year. Endemic: 100–299 new and relapse cases per 100.000 population per year. Highly endemic: 300–499 new and relapse cases per 100.000 population per year. Severely endemic: 500 or more new and relapse cases per 100.000 population per year [28]

***Risk of bias in included studies*** A mixed methods appraisal tool was used to assess the risk of bias and summarised in Table 3. More than half of the included studies (*n* = 10) ranked as high quality (100%) and addressed all the relevant criteria in the appraisal tool. This was followed by 80% (*n* = 5) and 60% (*n* = 2) of overall quality. Only one quantitative non-randomised study was identified as non-representative of the target population and did not report complete outcome data. The rest of the papers did not rank as high quality as they lacked clarity on the evaluation criteria.

**Table 3:** Risk of Bias in included studies

Studies	Criteria from the Mixed Methods Appraisal Tool																									Overall quality
	Qualitative					Quantitative randomised controlled trials					Quantitative non-randomised					Quantitative descriptive					Mixed methods					
	1.1	1.2	1.3	1.4	1.5	2.1	2.2	2.3	2.4	2.5	3.1	3.2	3.3	3.4	3.5	4.1	4.2	4.3	4.4	4.5	5.1	5.2	5.3	5.4	5.5	
Coreil et al. (2004)	1	1	2	1	1																					80%
Gao et al. (2015)	1	1	1	1	1																					100%
Gust et al. (2011)	1	1	1	1	1											1	1	2	2	1	1	1	1	1	1	60%
Hall et al. (2020)	1	1	1	1	1																					100%
Heyd et al. (2021)	1	1	1	1	1																					100%
Jacobson et al. (2017)	1	1	1	1	1																					100%
Kay et al. (2020)																1	1	2	1	1						80%
Nathavitharana et al. (2021)	1	1	1	1	1																					100%
Pekovic (1996)											0	1	0	1	1											60%
Rowe et al. (2005)	1	1	1	1	1																					100%
Seedat et al. (2014)	1	1	1	1	1																					100%
Skinner et al. (2013)	1	1	1	1	1																					100%
Sommerland et al. (2017)																1	1	1	1	1						100%
Spruikt et al. (2020)	1	2	1	1	1																					80%
Vasquez et al. (2022)	1	1	1	1	1																					100%
Williams (2015)	1	1	1	2	1																					80%
Yuan et al. (2023)																1	1	2	1	1						80%

Notes. 0 = No; 1 = Yes; 2 = Cant tell

1.1: Is the qualitative approach appropriate to answer the research question? 1.2: Are the qualitative data collection methods adequate to address the research question? 1.3: Are the findings adequately derived from the data? 1.4: Is the interpretation of results sufficiently substantiated by data? 1.5: Is there coherence between qualitative data sources, collection, analysis and interpretation? (2.1) Is randomization appropriately performed? (2.2) Are the groups comparable at baseline? (2.3) Are there complete outcome data? (2.4) Are outcome assessors blinded to the intervention provided? 2.5 Did the participants adhere to the assigned intervention? (3.1) Are the participants representative of the target population? (3.2) Are measurements appropriate regarding both the outcome and intervention (or exposure)? (3.3) Are there complete outcome data? (3.4) Are the confounders accounted for in the design and analysis? (3.5) During the study period, is the intervention administered (or exposure occurred) as intended? (4.1) Is the sampling strategy relevant to address the research question? (4.2) Is the sample representative of the target population? (4.3) Are the measurements appropriate? (4.4) Is the risk of nonresponse bias low? (4.5) Is the statistical analysis appropriate to answer the research question? (5.1) Is there an adequate rationale for using a mixed methods design to address the research question? (5.2) Are the different components of the study effectively integrated to answer the research question? (5.3) Are the outputs of the integration of qualitative and quantitative components adequately interpreted? (5.4) Are divergences and inconsistencies between quantitative and qualitative results adequately addressed? (5.5) Do the different components of the study adhere to the quality criteria of each tradition of the methods involved?

### The nature of stigma around TBI within TBI testing and treatment

Included papers have shown that TB stigma was a complex and multifactorial concept with six overlapping domains: public, anticipated, self, experienced, secondary, and structural. Different domains of stigma found co-occurring in the included papers, such as public and anticipated stigma [14, 29], anticipated and self-stigma [30, 31], and public and structural stigma [32]. 

The most commonly reported stigma type linked to engagement with testing and treatment practices was anticipated stigma [14, 29–31, 33–36]. The narrative summary of stigma types and their relevant constructs in the included papers are summarised below (Table 4).

**Table 4:** Nature of stigma around TBI within testing and treatment in the included studies

	Qualitative studies	Quantitative studies
Any TBI-related stigma, excluding secondary stigma	Anticipated stigma, [14, 29–31, 33–36] public stigma,[14, 30, 36–38] self-stigma, [14, 31, 32, 36, 39] enacted / experienced stigma, 14] structural stigma, [30, 32, 37] secondary stigma linked to TBI [40]	Anticipated stigma [41, 42], Others’ external TB stigma [40]
Discrimination or perceived poor treatment due to being linked to TBI	Negative representation in the media, [30] external TB stigma (witnessing others’ being stigmatised due to TB), [40] social exclusion,[14, 30, 31, 35] isolation [41, 43]	Discrimination [44]
Intersectional stigma or any other health-condition-related stigma linked to TBI	Racism, [30] social status,[36] ethnic stereotypes [30] immigration status, [30, 38, 43] HIV coinfection[14, 30, 33, 35]	HIV coinfection [41]

### The role/effect of TB stigma on taking part in TBI testing activities

**Fig. 2:**
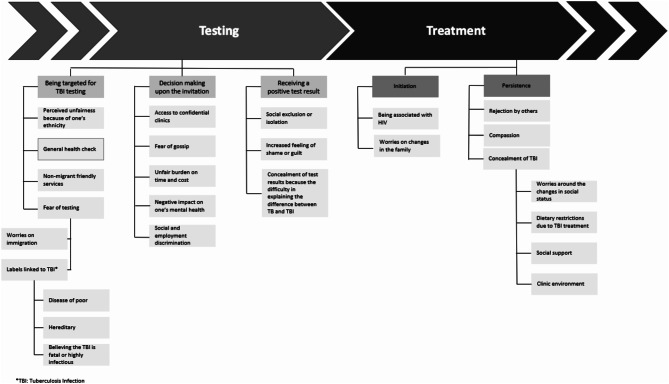
The role of TB stigma through TBI testing and treatment pathways

**Qualitative synthesis** The relationship between TB stigma and engagement with TBI testing activities was assessed under three sub-themes: being targeted for testing, decision-making upon the invitation and receiving a positive test result (Fig. 2).

***Being targeted for testing*** Being targeted for testing was linked to ‘*fear of testing*’ where people either worried about being associated with labels like having the ‘disease of poor’ or having a hereditary or fatal and highly infectious condition [37, 38]. Accordingly, self-stigma was the most common type of stigma found in the included papers when a person or group was targeted for testing [30, 37, 38]. People also worried about the testing negatively affecting their immigration status, fearing that they may be deported as a result of testing positive, either to reduce the spread of TB or due to the belief that society may not accept them [30, 37].

*“…So the people that were living with him said that if he goes to the doctor maybe they call immigration*,* they call the police*,* they call whoever*,* and finally the people that bring him to the hospital are the police because he went one day to buy food and on the way from the house to the shop he collapsed in the street and so someone called the police and the police came and the police took him to the hospital. And he died in the hospital three months later. It was too late. And many people have the same thing again*,* they don’t go to the hospital because they think that the hospital is going to call the police. So it stops people going. (Participant 11*,* age 31*,* male*,* Latin American community)”*

(UK, pg.5) [38].

In some studies, people felt that being targeted because of one’s ethnicity was unfair [30, 37]. Participants mentioned that due to globalisation and increased travel, anyone can be exposed to TBI, and targeting one specific ethnicity over others may result in racial stereotyping [37] or illness labelling [30]. Moreover, inaccessible or non-immigrant-friendly healthcare services make it harder for people to engage in testing [38]. It has been reported that having TBI testing as part of a general health check where multiple diseases are being checked would be perceived as less stigmatising or discriminatory by people [38].

*“…*,* people are going to – see the Pakistanis standing there*,* oh he might or she might have the TB and then you are going to create a stigma around that specific community*,* the Pakistanis or the Indians. Even when you are living over here*,* we are like the second citizens. Already there is some stigma around us .if any other stamp would be stamped on us*,* that would be very difficult …”*

(Australia, Pakistani female, *pg.356*) [37].

***Decision-making after receiving the invitation*** Decision-making following a testing invitation was related to five emerging sub-themes. Firstly, access to a confidential clinic consultation was found to increase involvement in TBI testing [30, 37], as it allowed people to avoid gossip [14] and possible discrimination in social and employment opportunities (e.g., loss of employment) [37]. Thus, decision-making after receiving an invitation was more linked to public and anticipated stigma subtypes [30, 37], which were also found to be interconnected with the experience of being targeted for testing, where the potential risk of self-stigma may be a driving factor. Individuals who felt targeted perceived this as being due to their ethnicity and reported feeling burdened by the associated time and cost, which may impact their decision to participate in testing [37]. Finally, individuals reported that decision-making regarding attending TBI testing may have a negative impact on mental health, leading to feelings of distress [37].

*“I think you should just think about yourself, for example, if I come to do the test and worry about what others may say about me, then it is hiding your own wound.”*

(Netherlands, Eritrean, pg.6) [14].

***Receiving a positive test result*** A positive TBI test result was linked to an increasing feeling of shame or guilt linked to self-stigma [39], as individuals believed they were the bringers of bad fortune upon themselves [39]. Furthermore, individuals reported that they concealed their TBI status because they found it difficult to explain the difference between TBI and active TB due to anticipating potential public stigma [14, 31, 38]. This association between TBI and active TB brought the fear of gossip, social exclusion or isolation demonstrating experienced stigma [30], potentially due to the infectiousness of active TB [14, 30, 31, 37].

*“From our background everything is kept a secret and admitting to people that you are suffering from this*,* it is a big stigma is*,* you could become an outcast. Normal things*,* like getting old*,* it’s fine*,* but TB again*,* it’s a bad thing. (P#3 Indian Female)”*

(Australia, pg.356) [37].

**Quantitative synthesis.** Only one study in South Africa explored stigma by using a validated questionnaire to measure others’ external stigma (witnessing another HCP experiencing TB stigma) on its potential impact on testing practices. The study found that increased perceptions of other healthcare worker’s stigma (e.g., witnessing someone mistreated because of TB) in the workplace decreased the probability of attending TB testing at an occupational health unit. Specifically, for each unit increase in perceived stigma, the probability of attending TB testing decreased by 0.214 (*B*: − 0.214, *p* < 0.001), after controlling for demographic and TB knowledge [40]. 

### The role/effect of TB stigma on treatment adherence

**Qualitative synthesis.** Several papers highlighted that fear of stigmatisation was a barrier to treatment initiation and adherence to TBI treatment [27, 37, 38, 42]. These effects were grouped under two subthemes: initiation and persistence (Fig. 2). However, one theme “being treated as equals” [32] as a result of good relationships with relatives and HCPs is reported to enhance treatment initiation, and motivation to continue and complete the TBI treatment.

*If personal rapport is not friendly the person will start to back off. I mean someone ill with TB has to be treated as if they are not sick*,* give them trust” (AI-07).*

(Dominician Republic, HCP, pg.8) [32].

***Treatment initiation*** Some participants expressed concerns about the diagnosis of TBI being associated with HIV, also known as other health-condition-related stigma [41]. There was reported concern that this link with HIV may lead to some families refusing TBI treatment [41]. However, in another study with patients living with HIV, no one named HIV-related stigma as a barrier to IPT adherence [35].

“*Most of people who have TB are my age*,* they have kids. They feel they would rather stay home because if they go to the clinic they will meet so and so who will start gossiping about them. They will label me as HIV as I have seen done to others. In the end children end up not getting treatment.*”

(South Africa, a parent of a child adherent to IPT, pg.196) [35].

Another study found that parents decided not to initiate IPT treatment for their children because of fears of stigmatisation and associated negative consequences such as losing custody of their children or having their partners leave them, suggesting structural stigma [32, 41]. These decisions not to initiate IPT were seen even in the best attempts of health system personnel explaining the risks involved [37]. 

***Treatment persistence*** Some parents spoke of having to overcome threats of isolation from their neighbours to keep their children on their IPT treatment, leading them to conceal the diagnosis as much as possible [38]. Adherence was also linked to stigma from healthcare professionals, with one study highlighting that “being treated as equals” [37] in the clinic setting enhanced treatment initiation, motivation to continue, and completion of TBI treatment. Many patients spoke about the need to conceal their TBI status due to fears their social status would be negatively affected [11, 25, 31, 37]. Having a private space in the clinics made it easier for people to conceal their TBI and was found as an important factor to facilitate persistence with treatment [27]. Dietary restrictions like not drinking alcohol during the treatment made it hard for people to conceal their treatment [11]. 

Some studies reported that even though people find it hard to continue their treatment due to anticipated stigma, and the potential negative reactions of others towards them, they still find ways to continue [11, 38]. Such as one study parents reported that their compassion towards their children kept them continuing their children’s treatment even though they may experience rejection by others [38]. Another study indicated people who disclosed their TBI status received mixed reactions from friends and relatives [14]. While some indicated receiving support from people who also took part in the education session on TBI helped with treatment adherence, others faced demotivating comments, including stories of harmful medication and social isolation, resulting in experienced stigma [14].

“*Despite their reaction, I wouldn’t want to stop my treatment. It might affect my feelings when they say things in front of me for the moment, but I don’t think of stopping my treatment. I am hoping to move to my own house, just to be away from their presence. But for now, until I finish my medicine, I just have to sit in my room. [II 22]”*

(Netherlands, participant on TBI treatment, p6) [14].

**Quantitative synthesis.** Four quantitative studies explored the effect of TB stigma on engagement with TBI treatment (in the USA, Uganda, China, and South Africa) [40–42, 44]. Stigma was measured by utilising validated questionnaires [40, 41] or those with internal consistency [42, 44]. Those questionnaires measured anticipated TB stigma [42, 44], witnessing others experiencing TB stigma [40] or stigma from various sources [41]. Two studies reported that TB stigma was found to be associated with suboptimal treatment activities [40, 41], supporting the results from the qualitative synthesis. One of these found that primary caregivers of children who were prescribed isoniazid preventive therapy (IPT) believed that taking a pill every day would lead to stigma from neighbours, thinking the child was sick or had TB, had increased odds of suboptimal adherence for the child (*OR*: 2.46, 95% *CI*: 1.10, 5.51 and *OR*: 1.75, 95% *CI*: 0.91, 3.37, respectively) [41]. In another study, higher perceptions of stigma from other healthcare workers in the workplace were associated with a reduced likelihood of receiving preventative TB treatment at the occupational health unit. Specifically, each increase in perceived stigma was linked to a 0.170 decrease in the probability of receiving IPT treatment (*B*: -0.170, *p* = 0.000). These effects persisted after controlling for demographic factors and TB knowledge [40]. In contrast, one study found that TB stigma was not related to treatment adherence (*B*: 0.23, *p* = 0.30) when controlling for socio-demographic factors and regimen-related characteristics [44]. Another study found an inverse relationship between stigma and treatment acceptance. In this study, involving college students from China perceived TB stigma was positively associated with TBI treatment acceptance (*OR* 1.03, 95% *CI* 1.00-1.08; *p* = 0.05), but only among male college students, (*OR* 1.07, 95% *CI* 1.02–1.12; *p* = 0.005) and no association found for female college students (*OR* 0.99, *95% CI* 0.94–1.05; *p* = 0.87).^42^

## Discussion

This systematic review was the first to explore the nature of TB stigma among people with TBI with its potential impact on engagement with TBI testing and treatment. The findings confirmed that the six types of TB stigma identified by the TB stigma handbook [12], seem to apply to TBI. The nature of TB stigma is complex and multifactorial [12], with its six domains (public, anticipated, self, experienced, secondary, and structural) overlapping. Overall, anticipated stigma appears to be the most experienced subtype among people with TBI. Interestingly, self-stigma was found to be a more frequently experienced subtype affecting engagement with TBI testing than with its treatment. TB stigma included being labelled as an outcast, being seen as infectious and being associated with HIV-related stigma. TB stigma was also linked to racism and ethnic stereotyping.

The results showed that stigma related to active TB was also reported in the context of TBI such as its infectiousness, indicating a carry-over of perceptions about active TB into TB infection, which affected motivation to engage with TBI testing and treatment. The fear of being labelled, judged, or experiencing discrimination associated with TB caused people to be reluctant to attend TBI testing or take treatment. Some people concealed their TBI status and treatment to avoid discrimination such as poor treatment, isolation, or exclusion in social settings. Thus, TB stigma is a complex psychosocial phenomenon that negatively affects relationships with oneself and others, hindering involvement in TBI testing and treatment activities to avoid psycho-socioeconomic losses.

Included studies highlighted that people find it hard to differentiate the concept of TB infection and its disease as separate entities. Being identified with TBI could result in similar labels as active TB, such as being considered infectious or facing discriminatory behaviours, akin to a person who has TB disease. Accordingly, the findings of this systematic review on TB stigma and its negative effects on engagement with TBI testing and treatment were similar to the results of a previous systematic review exploring the effects of stigma on mixed TB populations [11]. Even though people in this review were affected by TB infection not the disease, misplaced beliefs on the infectiousness of the condition or its non-treatability were common and drove perceived stigma related to TBI. Further, TB infection was associated with HIV, where HIV-related stigma may further add up to the burden of TB stigma and may result in a reluctance to engage in testing and treatment practices to avoid potential association with HIV. Therefore, it is important to consider how one’s sense-making of TBI as an illness may be linked to TB stigma and its impact on engagement with TBI care.

Disease prototypes are common ideas about diseases that may be mistaken from a medical perspective but influence behaviour [45, 46]. For example, a common TB prototype would be a severe illness that causes very visible symptoms such as cough, fever, night sweats, and weight loss. People who are told that they may have TB infection but do not have any of the signs and symptoms that they associate with TB (their TB prototype) may be less convinced of the necessity for testing or treatment [46]. It is also important to recognise the impact of TB stereotypes [47]. Illness stereotypes are commonly held ideas about the type of person who may get the disease [45]. TB is often perceived as a disease that affects those who are poor, unhygienic or with the human immunodeficiency virus (HIV) [11]. This stereotype may be stigmatising to the individual, as it may challenge how they see themselves and how they think others might see them [8]. This negative stereotype and its consequences for self-esteem may discourage engagement with TBI testing and treatment.

The TB prototypes and stereotypes described above are clearly misplaced especially in relation to TBI. However, if unchallenged they are likely to be a significant barrier to TBI testing and treatment. It was clear from our findings that offers of TBI testing and treatment often carry negative connotations for recipients, linked to TB stigma. Similarly, this review found that engagement in TBI testing was more commonly related to the self-stigma subtype compared to treatment, as receiving a positive diagnosis may prompt individuals to re-evaluate how they see themselves and how they will be viewed by others. Thus, the results of this systematic review show that TB stigma linked to active TB prototypes and stereotypes can result in increased anticipation or experience of social exclusion; this appears to affect people’s engagement in testing practices or follow-up on the TBI test results.

### Limitations

Even though this systematic review is the first review to explore the effects of TB stigma on testing and treatment engagement for TBI, there are several limitations. Some of the included studies did not explicitly define the TB type (infection vs. disease) or the subtypes of TB stigma, which resulted in authors making this judgement based on the available information within the paper. This process may result in missing relevant papers. Research mainly focused on treatment engagement while only 30% of included papers explored the effects of TB stigma on testing engagement. Further, even though this review included mixed TBI target groups, most of the participants were coming from immigrant populations. Therefore, this review may be more applicable to the experiences of immigrant populations. Followingly, we were unable to perform a subgroup analysis based on the year of the study or geographic location, as the limited number of studies prevented us from drawing meaningful comparisons regarding the experiences of stigma. Lastly, there were only four quantitative studies. Therefore, we were unable to conduct a meta-analysis to understand the quantitative impact of TB stigma on involvement in testing and treatment adherence. Further, most studies explored TB stigma as a secondary outcome, which limits the depth of available information on the effects of TB stigma among people with TBI, without specifically targeting TB stigma. Thus, especially the information coming from the qualitative studies may not be reported in greater detail within the papers, resulting in some of the themes found in the review not being explored in depth. There is a need for more research explicitly testing the impact of stigma and perceptions of TBI on testing and treatment engagement.

Research on stigma within TBI care is limited and current evidence mainly includes a relatively small number of qualitative studies. Also, the primary aim of these studies was to broadly review barriers to engaging with testing and treatment, rather than specifically explore the impact of TB stigma and its interactions with perceptions of TBI. Therefore, the information gathered from those studies is limited in nature. Future research could benefit from exploring the impact of TB stigma and perceptions of TBI as a primary aim of engagement with TBI care through quantitative studies. Further, those studies could also explore the possible moderators/mediators, such as necessity and concern beliefs, social exclusion, or self-regulation, to enhance our understanding of what to target in future interventions for TB stigma. Despite these limitations, our findings have implications for how TBI testing and treatment are communicated in practice and practice summarised below.

### Future implications

The WHO recommends targeting testing in high-risk populations such as specific immigrant populations where TB infection may be more commonly found [48]. However, this review demonstrated that immigrant or ethnic groups may view this specific targeting as a stigmatising and unfair approach towards themselves, which may put people off attending testing. Further, immigrant groups report being afraid of possible deportation. Thus, clinicians and policymakers need to be aware of the negative effects of targeting one specific group and may adapt to the use of less stigmatising or labelling language within their practices or policies. TBI care or testing being offered as a generic health check to patients, instead of a specific tailored approach which risks labelling people, may make it more acceptable among risk groups [38]. Alternatively, participants in these studies reported that confidential clinics may increase engagement with testing or treatment by reducing concerns about potential labelling and stigmatisation. Overall, HCPs and policymakers can further support this mission by providing stigma-free care zones for TBI by being aware of the ways which may stigmatise patients.

Population-wide strategies such as mass media campaigns as seen in HIV may help tackle TB stigma within TBI care. However, more research is needed on the impact of mass media campaigns. Research in HIV stigma suggests that these campaigns have a modest impact and are context-specific, suggesting alternative pathways, such as personalised approaches, are required [49]. Accordingly, changing prevalent public behaviour to not stigmatise people with TB/TBI status or not to exclude people with TBI in social contexts may not be possible. Therefore, as well as this, research can focus on identifying people’s perceptions of TB/TBI which may be further linked to TB stigma and on developing personalised interventions to reduce the impact of TB stigma on TBI care.

Interventions to encourage engagement with TBI testing and treatment are more likely to be effective if they take into account patient and public beliefs about TB and TBI. To convince patients of the necessity for testing and treatment, it is necessary to correct misplaced beliefs and concerns, including those related to stigma [50], as recommended by the National Institute for Health and Care Excellence (NICE) [51]. We also found that TB stigma is also experienced among HCPs and that concern of being stigmatised due to being linked to TB by other colleagues results in reduced involvement in occupational health activities and reluctance to be tested for TBI [40]. Therefore, interventions targeting TB stigma need to be integrated within the healthcare system to cover all ranges of TB-risk communities.

Preliminary research has shown that increased knowledge about TBI may be linked to higher reporting of TB stigma [52]. Similarly, this systematic review found similar findings, where even people who live with TBI patients who received TBI training from a TB nurse still demonstrated discriminatory behaviours towards people with TBI [14]. Thus, providing only knowledge-based interventions may not be able to address TB stigma in the context of TBI [52], as simply providing knowledge may not lead to a change in one’s perceptions of TBI [53]. It is important to move beyond the provision of information to communicate TBI in a way that changes misplaced beliefs. Several studies in other conditions have demonstrated the efficacy of this approach in enhancing adherence [54] and such approaches can be delivered by HCPs [55] but also through digital support [56]. 

## Conclusion

TB stigma is a significant factor that can hinder individuals’ engagement with TBI-related testing and treatment activities, leading to reluctance to seek treatment. Social representations of active TB with negative illness prototypes and stereotypes seem to be superposed on TBI. Consistently, TBI stigma is prevalent and may discourage engagement with TBI testing and treatment. There is a need for more behaviourally intelligent communication of TBI that convinces individuals of their personal necessity for TBI testing and treatment and mitigates their concerns by addressing beliefs about TBI including those that are perceived to be stigmatising.

## Electronic supplementary material

Below is the link to the electronic supplementary material.

Supplementary Material 1

## Data Availability

This study is a systematic review; no new datasets were generated or analysed. All data supporting the findings are derived from previously published articles, as cited in the manuscript.

## Abbreviations

TBI: Tuberculosis Infection
HIV: Human Immunodeficiency Virus
WHO: World Health Organization
