# Supplementary material for: A systematic review exploring the role of tuberculosis stigma on test and treatment uptake for tuberculosis infection
Source: BMC Public Health. 2025 Feb 14;25:628. doi: 10.1186/s12889-024-20868-0 (PMC11829483; doi:10.1186/s12889-024-20868-0)
Supplement: Supplementary file 1 — Supplementary Material 1 [file 12889_2024_20868_MOESM1_ESM.docx]

**Appendix A**

**Search Strategy**

| 1 | exp Latent Tuberculosis/ |
| --- | --- |
| 2 | Tuberc* or TB or LTBI or TBI |
| 3 | 1 OR 2 |
| 4 | "TB preventive therapy" or "TB preventative therapy" or "LTBI therapy" |
| 5 | TB or LTBI adj3 ( prophylaxis or chemoprophylaxis or chemoprevention) |
| 6 | “latent tuberculosis” or tuberculosis prevent* or inactive tuber* |
| 7 | exp Isoniazid/ |
| 8 | exp Rifampin/ |
| 9 | exp Rifamycins/ |
| 10 | exp Rifabutin/ |
| 11 | exp Pyrazinamide/ |
| 12 | exp Antitubercular Agents/ |
| 13 | IPT or 3HP or 1HP or H or RIF or RPT or PZA or TPT |
| 14 | 4 OR 5 OR 6 OR 7 OR 8 OR 9 OR 10 OR 11 OR 12 OR 13 OR screen* OR diagnos* |
| 15 | Engage* OR “medication adherence” [Mesh] OR adher* OR complian* OR uptake OR usage OR persist* |
| 16 | exp social stigma/ |
| 17 | Stigma* OR discrimin* OR “social discrimination” OR “social marginalization” OR “psychosocial impact” OR “socioeconomic impact” OR shame OR “social isolation” OR “social inclusion” OR prejudice OR isolation |
| 18 | 16 OR 17 |
| 19 | 3 AND 14 AND 15 AND 18 |
| 20 | 3 AND 14 AND 15 AND 18 **Filters: Humans, from 1964 - Current** |

*Note.* The search strategy was adapted based on the search operators of the included databases.
